# Assessing compatibility and viral fitness between poultry-adapted H9N2 and wild bird-derived neuraminidases

**DOI:** 10.1038/s41598-023-31653-1

**Published:** 2023-03-18

**Authors:** Anishia Wasberg, Inês R. Faria, Julia Bergholm, Philipp P. Petric, Ahmed Mostafa, Stephan Pleschka, Martin Schwemmle, Åke Lundkvist, Patrik Ellström, Mahmoud M. Naguib

**Affiliations:** 1 Zoonosis Science Center, Department of Medical Biochemistry and Microbiology, Uppsala University, Uppsala, Sweden; 2 Institute of Virology, Medical Center, University of Freiburg, Freiburg, Germany; 3 Faculty of Medicine, University of Freiburg, Freiburg, Germany; 4 Center of Scientific Excellence for Influenza Viruses, National Research Centre, Giza, Egypt; 5 Institute of Medical Virology, Justus Liebig University Giessen, Giessen, Germany; 6 German Center for Infection Research (DZIF),partner site Giessen-Marburg-Langen, Giessen, Germany; 7 Zoonosis Science Center, Department of Medical Sciences, Uppsala University, Uppsala, Sweden; 8 Present Address: Department of Biomedical Sciences and Veterinary Public Health, Swedish University of Agricultural Sciences, Box 7028, 750 07 Uppsala, Sweden

**Keywords:** Virology, Influenza virus, Pathogens

## Abstract

Exchange of viral segments between one or more influenza virus subtypes can contribute to a shift in virulence and adaptation to new hosts. Among several influenza subtypes, H9N2 is widely circulating in poultry populations worldwide and has the ability to infect humans. Here, we studied the reassortant compatibility between chicken H9N2 with N1–N9 gene segments of wild bird origin, either with an intact or truncated stalk. Naturally occurring amino acid deletions in the NA stalk of the influenza virus can lead to increased virulence in both mallard ducks and chickens. Our findings show extended genetic compatibility between chicken H9Nx gene segments and the wild-bird NA with and without 20 amino acid stalk deletion. Replication kinetics in avian, mammalian and human cell lines revealed that parental chH9N2 and rH9N6 viruses with intact NA-stalk replicated significantly better in avian DF1 cells compared to human A549 cells. After introducing a stalk deletion, an enhanced preference for replication in mammalian and human cell lines could be observed for rH9N2_Δ_(H6), rH9N6_Δ_ and rH9N9_Δ_ compared to the parental chH9N2 virus. This highlights the potential emergence of novel viruses with variable phenotypic traits, warranting the continuous monitoring of H9N2 and co-circulating subtypes in avian hosts.

## Introduction

Influenza A viruses (IAVs) predominantly circulate in wild aquatic birds and these avian IAVs (AIVs) can transmit to poultry. Poultry-adapted viruses can occasionally transmit to humans^1–3^. IAVs belong to the *Orthomyxoviridae* family with genetic material composed of segmented negative-sense RNA. The segmented genome allows for direct gene exchange between two or more IAVs co-infecting the same host cell, a mechanism termed reassortment, resulting in the emergence of novel subtypes^4–6^. The influenza virion contains eight gene segments that encode at least 10 proteins^7^ where the external domains of the two major surface glycoproteins, hemagglutinin (HA) and neuraminidase (NA) are used for subtyping different IAVs. There are 18 HA and 11 NA types described to date, whereas 16 HA and 9 NA are found in the avian population^4,8^; thus, theoretically, there are 144 possible HA–NA combinations. Over 100 out of these combinations have been reported to date, indicating extensive reassortment among different HA and NA genes. However, some subtypes are rarely found in nature, suggesting that some factors restrain particular HA–NA combinations^4,9^. Due to this genetic flexibility, IAVs are highly adaptable, able to transmit among a variety of host species and evade host immune responses^10,11^. Consequently, during this past century, four new IAVs causing global pandemics have emerged through reassortment^11,12^.

Since the late 1990s, low pathogenic AIV (LPAIV) of the H9N2 subtype has become one of the most widely detected AIV subtypes in poultry, posing a substantial economic burden to the poultry industry. In particular, this has affected countries in South East Asia and the Middle East^13^. Poultry-adapted H9N2 have been shown to preferentially bind human-like sialic acid receptors, while H9N2 viruses circulating in wild-bird species need prior adaptation in order to bind human sialic acid moieties efficiently^14–16^. However, recently H9N2 isolates derived from wild birds in China also displayed increased receptor affinity for human-like receptors^17^. Indeed, H9N2 has shown evidence of zoonotic transmission, predominantly causing mild upper respiratory tract infections in humans^18^. To date, reported human cases caused by the LPAIV H9N2 have been limited to the G1 and Y280 lineages, and no human-to-human transmission has been reported^5,16,19^. The H9N2 subtype tends to undergo extensive reassortment with co-circulating AIVs, including highly pathogenic AIV (HPAIV) H5Nx and H7Nx, as well as LPAIV H10N8^5^. Donation of internal genes from H9N2 has been verified in H5N1 and H7N9 viruses involved in several human cases of severe respiratory tract infections, some of which had lethal outcomes^20–23^. Recently, a core gene pool of the H9N2 internal genes has been defined which is suggested to act as a template for reassortment, revealing a strong association between reassortment events, geographical area and specific mutations^24^. Since its emergence, HPAIV H5N1 has acquired a NA-stalk deletion associated with increased virulence in avian hosts, such as mallards and chickens^25,26^, as well as mice^27,28^. In the course of transmission of AIVs from wild bird host species to domestic poultry, NA-stalk deletions have been associated with the early stages of this host transition. As of now, a variety of NA-stalk deletions of various lengths have been found in all classical NA subtypes^29,30^.

In this study, we used reverse genetics with the aim to assess the reassortment compatibility of the chicken-adapted H9N2 influenza virus A/Chicken/Egypt/S12568C/2016(H9N2) of the G1 lineage with NAs representing all 9 NA types (N1–N9) known to circulate in the wild bird population. In addition, we investigated whether a deletion of 20 amino acids (aa) in the NA stalk, commonly found in highly pathogenic influenza A/H5N1 viruses, affects this compatibility. Finally, we explored the replication kinetics of successfully rescued recombinant viruses on avian (DF-1), mammalian (MDCK-II), and human (A549) cell lines. 

## Results

### H9N2 is strongly overrepresented compared to other H9Nx combinations

To obtain a brief overview of the natural occurrence of various HA–NA combinations in IAVs from avian, mammalian and human hosts, all HA and NA sequences from these sources submitted to GISAID were collected and analyzed. The summarized sequence data in Table 1 demonstrate that combinations of HA and NA are not randomly distributed. Instead, certain HA/NA combinations were heavily overrepresented, whereas others were rare. The H9Nx combinations from all analyzed sequences revealed a significantly higher occurrence of N2 compared to other neuraminidases (97%, 14007/14255). Likewise, H9 isolated from domesticated birds were almost always found in combination with N2 (99%, 10789/10826). Focusing solely on the H9 isolates derived from humans, H9 was exclusively found in combination with N2 (100%, 86/86). In wild birds, H9 occurred in combination with several different NA, but the most common combination was H9N2 (92%; 1785/1936) in this group as well. There are likely many underlying factors behind the successful spread of H9N2 in poultry populations^31^. However, the fact that this was the most common combination also in wild birds prompted us to investigate whether genetic incompatibility between segments might be a cause of the overrepresentation of N2 in combination with H9.

**Table 1:** GISAID submitted HA and NA sequences from avian, mammalian and human hosts. Sequence data were accessed in Nov 2022.

	N1	N2	N3	N4	N5	N6	N7	N8	N9
H1	104,737	7508	116	12	26	14	14	56	60
H2	85	311	341	8	49	12	54	23	102
H3	230	141,015	55	10	23	448	26	4058	20
H4	42	263	48	27	26	2319	30	358	57
H5	11,653	2050	370	20	117	2520	32	3865	66
H6	710	1185	19	37	136	840	10	381	21
H7	450	551	1182	76	23	112	1413	50	3131
H8	2	5	2	267	2	1	2	2	0
H9	43	14,007	20	7	40	36	12	11	79
H10	94	72	215	112	175	78	914	169	55
H11	71	202	122	8	13	34	18	37	621
H12	24	28	22	55	301	10	11	16	12
H13	2	117	13	1	0	320	1	142	47
H14	0	2	23	2	11	6	3	2	0
H15	0	1	0	1	6	1*	1	1	10
H16	0	0	335	0	0	2	0	3	1

*Sequence information retrieved from the Influenza Virus Database (https://www.ncbi.nlm.nih.gov/genomes/FLU/Database/nph-select.cgi?go=database).

### NA cluster exclusively based on their subtype

**Figure 1:**
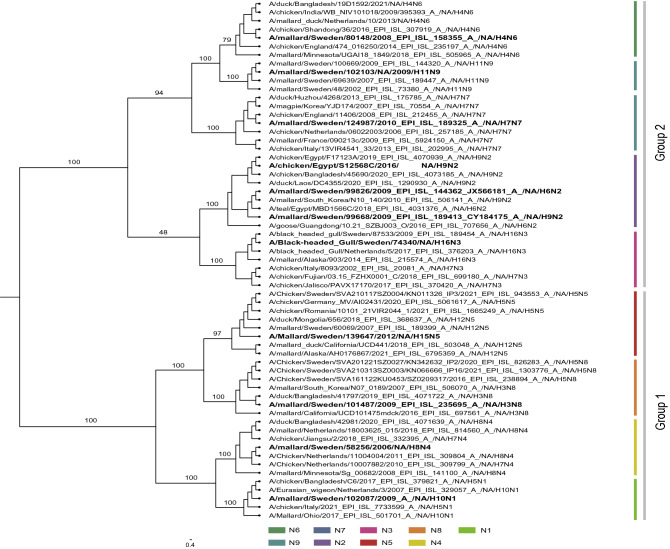
HYPERLINK "sps:id::fig1||locator::gr1||MediaObject::0" RAxML midpoint tree based on wild bird and poultry-adapted Neuraminidase sequences (N1–N9). The scale bar represents nucleotide substitutions per site, and branch labels show the bootstrap value in percentage. Virus isolates used in this study are highlighted in bold.

Earlier studies on the phylogenetic relationship between the NA types N1–N9 suggest that the NA genes form two distinct groups where group 1 contains N1, N4, N5 and N8, while group 2 contains N2, N3, N6, N7 and N9^32^. To confirm that the phylogenetic relationship between the different NA genes used in this study (including the NA segments used in the rescued reassortants) follows the same pattern, an extended phylogenetic analysis based on NA sequences of AIV subtypes from wild birds and poultry was performed (Fig. 1). The analysis showed the same phylogenetic clustering into two groups with the same internal relation between the NA segments. The phylogeny solely followed the NA classification since all N1–N9 clustered together independent of host and origin of isolation.

### Neuraminidase types N1–N9 from AIV of wild-bird origin exhibit genetic compatibility with poultry-adapted virus backbone

A possible explanation for the varying occurrence of the different HA/NA combinations shown in Table 1, particularly the extreme overrepresentation of H9N2 in poultry populations (99% of all combinations), could be that there is a genetic incompatibility between other NA segments and the H9 backbone virus, either in terms of physical incompatibility related to segment packaging during particle formation or functional incompatibility related to replication kinetics in the host cells. To study this in more detail, we generated recombinant viruses using the eight-plasmid reverse genetics system with the genetic backbone (PB2, PB1, PA, HA, NP, NS and M genes) from the poultry-adapted chH9N2 and NA gene segments from AIV of wild-bird origin representing all 9 NA types. All recombinant viruses rH9Nx (Table 2), including intact NA or short-stalk NA, were successfully rescued, indicating genetic compatibility between the poultry-adapted virus backbone and NA genes derived from wild-bird AIVs.

**Table 2:** List of generated recombinant rH9Nx viruses with and without stalk deletion. Letters m = mallard and g = black-headed gull denotes the host origin. All recombinant viruses have a gene backbone derived from the chH9N2 (PB2, PB1, PA, HA, NP, NS and M) together with a wild bird derived NA-gene.

Parental viruses	Recombinant viruses with intact NA	Recombinant viruses with NA stalk deletion
Backbone × NA-gene		
chH9N2 × mH10N1	rH9N1	rH9N1_Δ_
chH9N2 × mH6N2	rH9N2_(H6)_	rH9N2_Δ(H6)_
chH9N2 × mH9N2	rH9N2_(H9)_	rH9N2_Δ(H9)_
chH9N2 × gH16N3	rH9N3	rH9N3_Δ_
chH9N2 × mH8N4	rH9N4	rH9N4_Δ_
chH9N2 × mH15N5	rH9N5	rH9N5_Δ_
chH9N2 × mH4N6	rH9N6	rH9N6_Δ_
chH9N2 × mH7N7	rH9N7	rH9N7_Δ_
chH9N2 × mH3N8	rH9N8	rH9N8_Δ_
chH9N2 × mH11N9	rH9N9	rH9N9_Δ_

### The rH9N2(H6), rH9N4, rH9N5, rH9N7 and rH9N8 viruses replicate significantly better in human A549 cells compared to the parental chH9N2

**Figure 2:**
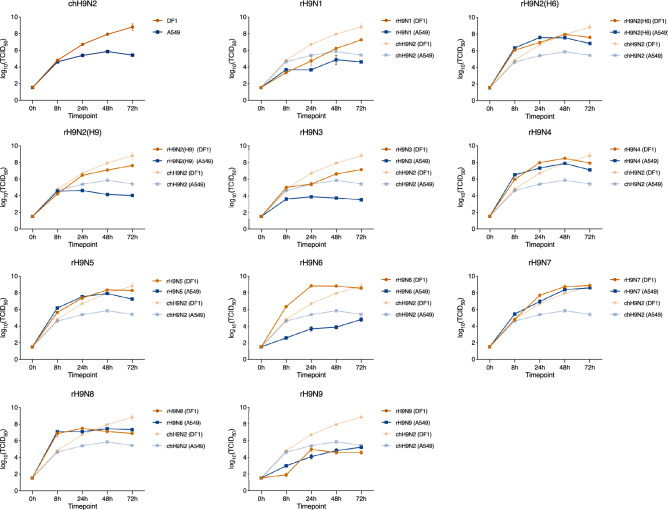
Replication efficiency of the rH9Nx reassortants with intact neuraminidase stalks in DF-1 (orange) and A549 (blue). The log_10_(TCID_50_) value at time point 0 h is based on the starting TCID_50_ value. Error bars represent the standard error of the mean.

To study potential differences in replication kinetics after the introduction of NA segments from AIV of wild bird origin in the parental chH9N2 backbone, avian (DF-1), mammalian (MDCK-II) and human (A549) cell lines were infected with the chH9N2 and the generated recombinant viruses. Most of the viruses were able to establish infection in all three cell lines, with varying efficiencies (Fig. 2). The parental chH9N2 demonstrates significantly higher replication in DF-1 cells compared to A549, which is well in accordance with the fact that chH9N2 is poultry-adapted. Recombinant reassortants rH9N1, rH9N2(H9) and H9N9 did not efficiently replicate in DF-1 cells and replicated at a significantly lower rate compared to the parental chH9N2 (Table 3). In contrast, at early timepoints, rH9N4, rH9N6 and rH9N7 showed significantly higher replication in the avian DF-1 cells compared to chH9N2. Recombinant reassortants rH9N1, rH9N2(H9), rH9N3 did not efficiently replicate in A549 cells (Fig. 2). Additionally, rH9N6 replicated significantly less efficient in both A549 and MDCK-II (Fig. 2, Table 3, Suppl. Table 6). In the A549 cells, rH9N2(H6), rH9N4, rH9N5, rH9N7 and rH9N8 displayed significantly better replication efficiency compared to the parental chH9N2 (Fig. 2, Table 3). When comparing the replication efficiencies of the rH9Nx viruses in DF-1 and A549 cells, most of the viruses replicate significantly better in DF-1 cells (Suppl. Table 3). These results suggest that introducing the different NA (N1–N9) segments into the poultry-adapted H9N2 virus backbone significantly impacts replication kinetics of the recombinant viruses, resulting in either increased or reduced replication efficiency depending on NA segment and cell line. In some cases, the reassortant viruses displayed increased preference for replication in human cells.

**Table 3:** List of p-values generated from Tukey multiple comparison of the replication kinetics data, comparing the mean TCID_50_ between rH9Nx with intact neuraminidase stalk and parental chH9N2 virus in both DF-1 and A549 cells. P-values less than 0.05 were considered significant. Bold p-values indicate significantly higher mean TCID_50_ value at a given timepoint. Underlined p-values indicate significantly lower TCID_50_ at a given timepoint. Non-significant comparisons are denoted ‘ns’.

	DF-1					A549			
	8 h	24 h	48 h	72 h		8 h	24 h	48 h	72 h
**rH9N1**	0.0001	ns	0.0008	ns	**rH9N1**	0.0231	0.0002	ns	0.0345
**rH9N2**_**(H6)**_	**0.0341**	ns	ns	ns	**rH9N2**_**(H6)**_	**0.0009**	**0.0001**	**0.0055**	**0.0137**
**rH9N2**_**(H9)**_	0.0019	ns	0.0038	ns	**rH9N2**_**(H9)**_	ns	ns	0.0100	0.0046
**rH9N3**	ns	ns	ns	ns	**rH9N3**	0.0163	0.0002	0.0019	0.0085
**rH9N4**	**0.0052**	**0.0005**	**0.0166**	ns	**rH9N4**	**0.0007**	**0.0001**	**0.0092**	**0.0037**
**rH9N5**	**0.0013**	ns	ns	ns	**rH9N5**	**0.0016**	** < 0.0001**	**0.0045**	**0.0041**
**rH9N6**	**0.0052**	**0.0108**	**0.0108**	ns	**rH9N6**	0.0005	0.0495	0.0068	ns
**rH9N7**	ns	**0.0182**	**0.0078**	ns	**rH9N7**	**0.0340**	**0.0418**	**0.0026**	**0.0004**
**rH9N8**	ns	ns	0.0008	ns	**rH9N8**	**0.0004**	**0.0002**	**0.0136**	**0.0031**
**rH9N9**	0.0122	ns	0.0067	0.0063	**rH9N9**	0.0014	ns	ns	ns

### The rH9N2_Δ_ (H6), rH9N6_Δ_ and rH9N9_Δ_ display increased replication in mammalian cell lines 

**Figure 3:**
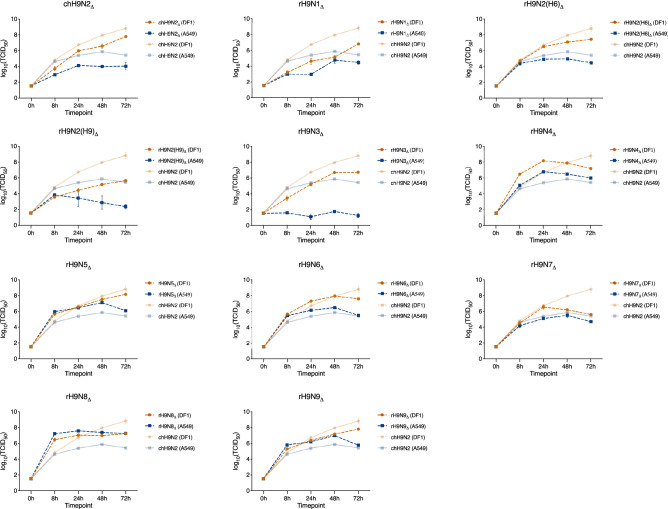
Replication efficiency of the rH9Nx reassortants with a 20 aa neuraminidase stalk deletion in DF-1 (orange) and A549 (blue). The log_10_(TCID_50_) value at time point 0 h is based on the starting TCID_50_ value. Error bars represent the standard error of the mean (SEM).

In addition to assessing the replication kinetics of generated recombinant reassortants with intact NA from AIV of wild bird origin, the replication capacities of the viruses with 20 aa NA stalk truncations was assessed. The replication kinetics of the viruses with NA-stalk deletions were similar to that observed for the viruses with an intact NA-stalk, albeit with generally reduced replication in all cell lines (Fig. 3). However, rH9N5_Δ_, rH9N6_Δ,_ rH9N8_Δ_ and H9N9_Δ_ displayed significantly better replication in A549 cells compared to chH9N2. The rH9N4_Δ_ instead, displayed a significant replication increase in DF-1 cells at early timepoints (8 h and 24 h) compared to chH9N2 (Table 4). When comparing the replication of the rH9Nx with 20 aa stalk deletions in DF-1 and A549 cells, for the majority of viruses, a significantly better replication in DF-1 cells could be observed (Suppl. Table 4). In MDCK-11 cells, rH9N2_Δ_(H6) and rH9N6_Δ_ and rH9N9_Δ_ displayed increased replication compared to their homologous counterparts with intact NA stalks, although a significant difference was only observed for rH9N6_Δ_ (Suppl. Table 7). Additionally, rH9N6_Δ_ and rH9N9_Δ_ displayed increased replication in A549 cells compared to their homologs with intact NA-genes (Suppl. Table 5). This was also the case for rH9N9_Δ_ in DF-1 cells.

**Table 4:** List of p-values generated from Tukey multiple comparison of the replication kinetics data, comparing the mean TCID_50_ between rH9Nx with neuraminidase stalk-deletion and parental chH9N2 virus in both DF-1 and A549 cells. P-values less than 0.05 were considered significant. Bold p-values indicate significantly higher mean TCID_50_ value at a given timepoint. Underlined p-values indicate significantly lower TCID_50_ at a given timepoint. Non-significant comparisons are denoted ‘ns’.

	DF-1					A549			
	8 h	24 h	48 h	72 h		8 h	24 h	48 h	72 h
**chH9N2**_**Δ**_	ns	0.0033	ns	ns	**chH9N2**_**Δ**_	0.0097	0.0046	0.0084	ns
**rH9N1**_**Δ**_	ns	ns	0.0008	ns	**rH9N1**_**Δ**_	0.0016	0.0035	ns	ns
**rH9N2**_**(H6) Δ**_	ns	ns	0.0013	ns	**rH9N2**_**(H6) Δ**_	ns	0.0451	ns	0.0321
**rH9N2**_**(H9) Δ**_	0.0008	0.0348	< 0.0001	0.0441	**rH9N2**_**(H9) Δ**_	ns	ns	ns	0.0209
**rH9N3**_**Δ**_	ns	0.0270	0.0003	ns	**rH9N3**_**Δ**_	0.0002	0.0219	0.0003	0.0067
**rH9N4**_**Δ**_	**0.0002**	**0.0007**	ns	ns	**rH9N4**_**Δ**_	ns	**0.0014**	ns	ns
**rH9N5**_**Δ**_	**0.0051**	ns	ns	ns	**rH9N5**_**Δ**_	**0.0217**	**0.0006**	**0.0211**	**0.0376**
**rH9N6**_**Δ**_	ns	**0.0231**	ns	ns	**rH9N6**_**Δ**_	**0.0003**	**0.0372**	**0.0072**	ns
**rH9N7**_**Δ**_	**0.0239**	ns	0.0074	0.0319	**rH9N7**_**Δ**_	ns	ns	ns	0.0354
**rH9N8**_**Δ**_	ns	ns	ns	ns	**rH9N8**_**Δ**_	**0.0027**	**< 0.0001**	**0.0106**	**0.0011**
**rH9N9**_**Δ**_	ns	ns	0.0333	ns	**rH9N9**_**Δ**_	**0.0132**	**0.0333**	**0.0339**	ns

### NA stalk deletion increases NA-activity in rH9N1_Δ_, rH9N3_Δ_ and rH9N4_Δ_

**Figure 4:**
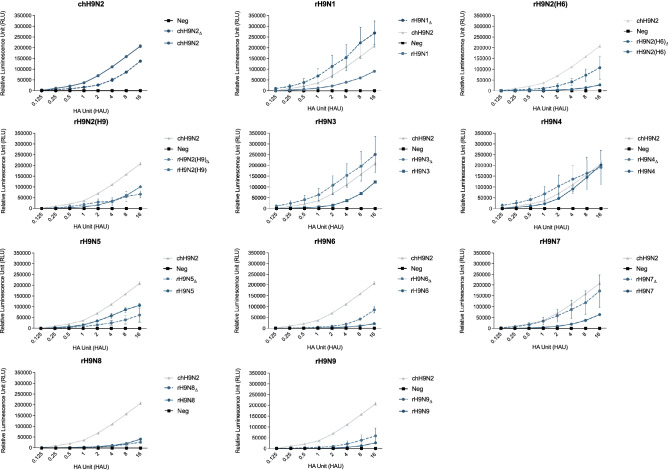
NA activity of the rH9Nx reassortants with and without a 20 aminoacid stalk deletion. Graph demonstrates the Relative Luminescence Unit at given HAU titers. Errorbars represent the standard error of the mean (SEM).

The functional balance between the HA and NA glycoproteins has repeatedly been reported to be crucial for viral entry and establishment of infection^33^. The NA cleavage activities were assessed for all recombinant reassortants to investigate how this factor relates to the observed replication kinetics of the viruses (Fig. 4). The NA-activities were significantly reduced in all the reassortants with intact NA compared to the parental chH9N2, except for rH9N4, which also had a decreased activity, although not significant (Suppl. Table 8). Similarly, the NA-activities of the stalk-deletion reassortants were generally reduced compared to the chH9N2, except for the rH9N1_Δ_, rH9N3_Δ_ and rH9N4_Δ_ where the activities instead increased. Comparing the NA activities between recombinant reassortants with intact NA stalks to their homologous counterparts with stalk-deletions revealed that the stalk deletion increased the NA activity significantly (at higher HAU) in rH9N1_Δ_, rH9N2(H6)_Δ_, rH9N3_Δ_, rH9N6_Δ_, and rH9N7_Δ_. In contrast, the stalk deletions reduces the NA activity of rH9N2(H9)_Δ_, rH9N5_Δ_, and rH9N8_Δ_ (Suppl. Table 9). Although we observed differences in NA activities between the recombinant reassortants, there were no obvious correlation between NA-activity and replication kinetics in our experimental setting.

## Discussion

Reassortment events play an essential role in the diversity of AIVs through the genetic exchange between co-circulating viruses of different subtypes^34^. Since the 1990s, the LPAIV subtype H9N2 has spread throughout the world’s poultry populations and has been found in an increasing number of countries. Frequently recorded reassortment events between H9N2 and other co-circulating AIV subtypes necessitate further research on potential restrictions for reassortment of this AIV subtype. Four main factors contributing to genetic incompatibility between different viral subtypes have been suggested to restrict the genesis of reassortants: (1) incompatibility between packaging signals of gene segments from various IAV strains, (2) segment mismatch referring to incompatible protein–protein interactions, (3) transcription/replication incompatibility between the RNA-dependent RNA polymerase and viral gene segments, or (4) inhibitory effects of one IAV subtype on another^35,36^. In addition to these mechanisms, virus-extrinsic spatiotemporal factors also play a role in restricting reassortment between co-infecting viruses^37^. Altogether, the factors dictating efficient reassortment are likely more complex and extend beyond the mentioned mechanisms; thus, conditions favoring efficient reassortment remain to be elucidated. Considering the wide spread of H9N2 in poultry species, we investigated the genetic compatibility between chicken H9N2 and wild bird-derived AIV neuraminidases (N1–N9) with and without a 20 aa deletion in the stalk region of the NAs.

By using a reverse genetics system, rH9Nx reassortants with NA gene segments representing all neuraminidase types (N1–N9) found in the AIV gene pool could be successfully rescued. This is in agreement with the outcome of our in silico analysis (Table 1), which demonstrated previous records of naturally occurring combinations, including H9N1–H9N9 subtypes, although the occurrence of any other subtype than H9N2 was very low, especially in poultry species. Our findings indicate genetic compatibility between the parental chH9N2 and NAs from all other AIV subtypes of wild bird origin, implying that genetic or physical incompatibility with other NA segments in the influenza A virion is not the restricting factor resulting in the overrepresentation of H9N2 in poultry. Both HA and NA demonstrate hypervariability in their aa sequences and within a subtype, up to 20% aa sequence diversity has been observed^7,38^. Hence, the NA diversity needs to be taken into consideration. In this study, we used only one wild-bird-derived AIV NA segment as representative of each NA type in the reassortment experiments, except for N2 where two different segments were used. It is possible that not all NA segments of every NA type is compatible with the chH9N2 backbone and future studies with a wider diversity within each NA type might reveal such differences. On the other hand, the fact that all tested NA segments generated successfully resuscitated virus particles suggest a broad “tolerance” to genetically distant NA segments within this backbone. Adaptation to poultry species and other hosts can be another factor limiting the spread of the other H9Nx subtypes. Therefore, we have assessed the impact of NA exchange on the replication kinetics in different cell lines harboring different structures of sialic acid receptors, including a chicken cell line (DF-1) expressing the avian-like receptor (α2,3 SA)^39,40^, a human cell line (A549) and another mammalian cell line (MDCK-II) that both harbor human-like receptors (α2,6 SA) as well as the avian-like receptor^41,42^. The replication kinetics results of the intact NA-reassortants indicated replication capacity in all three cell lines, with a significantly higher replication in DF-1 cells compared to A549. Notably, rH9N1, rH9N2(H9), rH9N3, rH9N8 and rH9N9 demonstrated higher replication in MDCK-II cells at 72 h post-infection compared to DF-1 and A549.

As demonstrated by several groups, the length of the NA-stalk affects the biological characteristics of influenza viruses; truncated NAs are widespread among AIVs and associated with increased virulence in chickens^43–45^. Thus, we assessed the genetic compatibility and virus replication kinetics of the N1–N9 reassortants after introducing a 20 aa deletion in the NA stalk region. Genetic compatibility was not impacted by the NA-stalk deletion considering reassortants with all NA subtypes (N1–N9) were successfully rescued. The majority of the reassortants with a stalk deletion displayed increased replication in both MDCK-II and A549 compared to DF-1, although with a lower replication rate compared to the intact NA-reassortants. Interestingly, rH9N2_Δ_(H6), rH9N6_Δ_ and rH9N9_Δ_ viruses showed an increased replication in MDCK-II and A549 compared to their homologous counterparts with an intact NA-gene. Collectively, our replication kinetics observations imply that changing NA has no significant impact on establishing infection in either DF-1, MDCK-II, or A549. However, a truncated NA-stalk appears beneficial in terms of replication kinetics in mammalian cells.

The HA and NA have opposite functions and thus have to work cooperatively to facilitate movement on cell surfaces and ultimately efficient attachment and viral entry. A suboptimal NA activity in relation to the activity of HA, may obstruct or aggravate the ability of the virus to infect a cell^33,46^. In this study we assessed the NA-activity for rH9Nx reassortants with and without stalk deletion. Although significant differences were observed in NA activities between the recombinant reassortants, these differences did not show any consistent correlation to the replication kinetics of the viruses. We have not studied if and how the introduction of new NA segments into the chH9N2 backbone virus affect the relative abundance of HA and NA molecules in the virus envelope. Hence, the effect on replication kinetics after introduction of a NA segment with a different NA activity compared to the parental virus, might have been masked by an altered relative abundance of HA and NA in the reassortant virus.

The NA-stalk region varies considerably between subtypes and NA-stalk deletions is commonly found in different avian influenza viruses^29,30,47^. Studies have shown that NA-stalk deletion can have varying effects on AIVs^48–51^, however, stalk deletions are commonly associated with adaptation to poultry species^30,43,49^. In our study, all recombinant viruses with NA stalk deletion were successfully rescued indicating that the 20 amino acid deletion is not detrimental to virus rescue and replication. All recombinants, with stalk deletion, generated in the study had significant lower or similar replication in DF1 cells at 48 h and 72 h post infection (Table 4). This correlates with what was previously published by Arai et al., where they showed that 21 amino acid stalk deletion is associated with increased growth in human cell line compared to a reduction in replication kinetics in chicken cell line^46^. Our and previous findings show that the effects of NA-stalk deletions in avian viruses are complex and depend on a variety of factors.

In conclusion, remarkable genetic compatibility was found between the chicken H9N2 backbone and N1–N9 from AIV of wild bird origin where the NA stalk length did not have any impact. However, reassortant viruses with truncated NA-stalk revealed increased replication kinetics in mammalian cell lines. These findings provide insights into the potential emergence of H9Nx viruses in nature and how they behave in different cell hosts, which calls for continuous monitoring of reassortant events at the domestic-to-wild-bird interface. Further studies of infection efficiency and replication kinetics of these viruses in chickens are needed to better estimate the role of the different NA segments in vivo.

## Materials and methods

### In silico analysis

To investigate the natural occurrences of the HA and NA gene combinations in AIVs reported among avian species (16 HA and 9 NA) with an emphasis on H9, all available influenza sequences from fully sequenced HA and NA genes on the Global Initiative on Sharing All Influenza Data (GISAID) database were retrieved (accessed: 1st of November 2022) and summarized in Table 1. HA–NA combinations not found on GISAID were cross-checked on the Influenza Virus Database to ensure complete coverage of all available influenza sequences. Additionally, a phylogenetic analysis of N1–N9 sequences from wild birds and poultry hosts was performed. Briefly, NA sequences were downloaded from the GISAID database and aligned using MAFFT (MAFFT online service, https://mafft.cbrc.jp/alignment/server/). In Geneious Prime v.2022.1.1 phylogenetic tree was generated using the RAxML (Randomized Axelerated Maximum Likelihood v8.2.11) plug-in using nucleotide model GTR GAMMA. Finally, the phylogenetic tree was visualized using FigTree version 1.4.4 (http://tree.bio.ed.ac.uk/software/figtree/).

### Viruses

Ten LPAIV viruses resembling all AIV NAs (N1–N9) circulating in wild birds, including two N2 genes from mallard H9N2 and H6N2 (Supplementary Table 1), were obtained from Jonas Waldenstöm (Linneaus University, Sweden) and used in this study. Viruses were initially isolated from wild birds at the Ottenby Bird Observatory (Öland, 56.19765; 16.39939, Sweden). In addition, the G1-like lineage chicken LPAIV H9N2 influenza virus A/Chicken/Egypt/S12568C/2016 (hereafter chH9N2) was rescued via the eight-plasmid reverse genetics system described previously^52^.

### Plasmid construction and cloning

In brief, viral RNA was extracted using QIAamp^®^ viral RNA kit (Qiagen, Hilden, Germany) according to the manufacturer’s protocol. Following extraction, NA gene segments from viruses in Supplementary Table 1 were amplified using a high-fidelity Superscript IV One-Step RT-PCR kit (Thermo Fisher Scientific, MA, USA) with specific forward and reverse primers, including segment-specific sequences and BsmBI or BsaI restriction sites at both 3′ and 5′ ends (Supplementary Table 2). Subsequently, pHW2000 expression plasmid and PCR products were digested using either BsmBI or BsaI (New England Biolabs, MA, USA) restriction enzymes and then ligated together in a 3:1 insert/plasmid ratio using T4 DNA ligase (New England Biolabs, MA, USA). The ligated pHW2000-NA plasmids were transformed into competent DH5α *E. coli* (Thermo Fisher Scientific, MA, USA) using the standard heat shock method. After the transformation step, selected clones were verified by purification of plasmid DNA using the Zyppy^®^ plasmid miniprep kit (Zymo Research, CA, USA) and subsequent sequencing at (Eurofins Genomics, Ebersberg, Germany) to ensure correct gene orientation and sequence. Eleven expression plasmids containing the different NA-gene segments were constructed. Additionally, 11 pHW2000 plasmids containing a 20 aa deletion in the NA-gene were constructed using Q5^®^ Site-Directed Mutagenesis Kit (New England Biolabs, MA, USA). Specific primers (Supplementary Table 2) for N1–N9 were designed to delete 20 aa at nucleotide positions 148–207.

### Cell culture

Madin–Darby canine kidney cells (MDCK-II), Human embryonic kidney cells (HEK 293T), and Adenocarcinomic human alveolar basal epithelial cells (A549) were cultured in flasks containing Dulbecco Modified Eagle Medium (DMEM, Gibco™, Life Technologies, UK) including 5% (v/v) fetal bovine serum (FBS, Gibco™, Life Technologies, UK), and 1% (v/v) Antibiotic–Antimycotic (Anti-Anti, Gibco™, Life Technologies, UK). Additionally, Chicken embryo fibroblast (DF-1) cells were cultured in flasks containing 50% Ham’s F12 nutrient mix (F12), 50% Iscove Modified Dulbecco Media including 5% (v/v) FBS and 1% (v/v) Anti-Anti. Cells were kept at 37 ºC with an air circulation containing 5% CO_2_. Cells were provided by Friedrich-Loeffler-Institut, Greifswald-Insel Riems, Germany.

### Rescue protocol and generation of recombinant reassortant viruses

A “7 + 1” reverse genetics approach was used previously described by Hoffman et al., including seven gene segments (PB1, PB2, PA, HA, NP, NS, and M) from the parental chH9N2 and one of the 22 generated NA gene segments (10 intact and 10 with a 20-aa deletion in the stalk region). As a positive control, parental chH9N2 was rescued using all eight plasmids of this virus. Virus rescue trials were performed in co-cultures of 293T and MDCK-II cells at a 1:3 ratio cultivated in 6-well plates. Prior to transfection, cells were serum-starved for 1 h at 37 °C in Opti-MEM medium (Gibco™, Life Technologies, UK). In brief, cells were transfected with 500 ng of 7 + 1 plasmids using Lipofectamine™ 3000 transfection reagents (Invitrogen by ThermoFisher Scientific, CA, USA) according to standard procedure. At 24 h post-transfection, the media was changed to 3 ml of infection media per well containing Opti-MEM (Gibco™, Life Technologies, UK) supplemented with 0,2% (v/v) Bovine Serum Albumin solution 35% (BSA, MpBio™, CA, USA), 0.5 µg/µl TPCK-treated Trypsin (Sigma-Aldrich, MO, USA), and 100 I.U./ml Penicillin and 100 μg/ml Streptomycin (Gibco™, Life Technologies, UK). Supernatants were collected at two time points, 48 h and 72 h, and subsequently inoculated in specific pathogen-free (SPF) 10-day-old embryonated chicken eggs. The allantoic fluid was harvested 72 h post-inoculation and viral content was confirmed by qPCR using AgPath-ID™ One-Step RT-PCR kit (Applied Biosystems™ by Thermo Fisher Scientific, CA, USA) using primers targeting N1–N9 developed by Hassan et al.^53^.

### In vitro replication kinetics 

The replication kinetics of the recombinant reassortant viruses were assessed in avian (DF-1), mammalian (MDCK-II) and human (A549) cells. Trials to obtain plaques and calculate the MOI were not successful for all generated recombinant viruses using different concentration of either microcrystalline cellulose Avicel or agarose as overlay for plaque formation. Therefore, all viruses were titrated in MDCK-II cells and normalized to TCID_50_ (3.16 × 10^2^/ml). Cells were seeded in 24-well plates and all infections were performed in triplicate wells. At 1 h post-infection, the media was changed to infection media containing DMEM (Gibco™, Life Technologies, UK) supplemented with 0.2% (v/v) Bovine Serum Albumin solution 35% (BSA, MpBio™, CA, USA), 0.5 µg/µl TPCK-Trypsin with a concentration of 2.5 mg/ml (Sigma-Aldrich, USA), and 100 I.U./ml Penicillin and 100 µl/ml Streptomycin (Gibco™, Life Technologies, UK) and the cells were further incubated for 72 h. At timepoints 8 h, 24 h, 48 h and 72 h post-infection, cells were checked for cytopathic effects and supernatants were collected. Ultimately, viral RNA was extracted from the supernatant, and the viral content was assessed by qPCR using AgPath-ID™ One-Step RT-PCR kit (Applied Biosystems™ by Thermo Fisher Scientific, CA, USA). Statistical analyses comparing the replication kinetics of the rescued viruses were performed by two-way repeated-measures ANOVA followed by Tukey multiple pairwise comparison using Graphpad Prism (version 9.5.1).

### Neuraminidase activity

The neuraminidase enzymatic activity of the recombinant reassortants was assessed by cleavage of the soluble substrate NA-XTD using the NA-XTD™ Influenza Neuraminidase Assay kit (Applied Biosystems™ by Thermo Fisher Scientific, CA, USA) following the manufacturers protocol. All viruses were normalized to an equivalent hemagglutinin unit of 32 HAU. In brief, viruses were diluted in a twofold dilution series with the assay buffer followed by addition of substrate and a 30 min room temperature incubation. Thereafter, an assay accelerator was added and the chemiluminescence was measured using infinite^®^ M200 plate reader (Tecan, Männedorf, Switzerland). A two-way ANOVA using GraphPad Prism was done to compare the NA-activity between the viruses.

## Supplementary Information

Supplementary Information.

## Data Availability

All data generated in this study are included in this published article and its Supplementary Information files.
