# Supplementary material for: Assessing compatibility and viral fitness between poultry-adapted H9N2 and wild bird-derived neuraminidases
Source: Sci Rep. 2023 Mar 18;13:4476. doi: 10.1038/s41598-023-31653-1 (PMC10024770; doi:10.1038/s41598-023-31653-1)
Supplement: Supplementary file 1 — Supplementary Information. [file 41598_2023_31653_MOESM1_ESM.docx]

Supplementary Material

Assessing compatibility and viral fitness between poultry-adapted H9N2 and wild bird-derived neuraminidases

Anishia Wasberg^1^, Inês R. Faria^1^, Julia Bergholm^1^, Philipp P. Petric^2,3^, Ahmed Mostafa^4^, Stephan Pleschka^5,6^, Martin Schwemmle^2,3^, Åke Lundkvist^1^, Patrik Ellström^7^, Mahmoud M. Naguib^1*^

^1^Zoonosis Science Center, Department of Medical Biochemistry and Microbiology, Uppsala University, Sweden

^2^Institute of Virology, Medical Center, University of Freiburg, Freiburg, Germany

^3^Faculty of Medicine, University of Freiburg, Freiburg, Germany

^4^Center of Scientific Excellence for Influenza Viruses, National Research Centre, Giza, Egypt

^5^Institute of Medical Virology, Justus Liebig University Giessen, Giessen, Germany

^6^ German Center for Infection Research (DZIF), partner site Giessen, Germany

^7^Zoonosis Science Center, Department of Medical Sciences, Uppsala University, Uppsala, Sweden

*** Correspondence:**Mahmoud M. Naguib
Mahmoud.naguib@imbim.uu.se

# Supplementary Figures

Supplementary Figure . Replication efficiency of the rH9Nx reassortants with intact and deletion neuraminidase stalks in MDCK-ll cells. The log_10_(TCID_50_) value at time point 0h is based on the starting TCID_50_ value. Error bars represent the standard error of the mean.

Supplementary Figure . Replication efficiency of the rH9Nx reassortants in DF-1 (orange) and A549 (blue). Each graph displays the mean TCID_50_ for both recombinant reassortants with and without stalk deletion and the parental chH9N2 virus. The log_10_(TCID_50_) value at time point 0h is based on the starting TCID_50_ value. Error bars represent the standard error of the mean.

#

# Supplementary Tables

Supplementary Table 1. List of wild-bird derived viruses used in this study.

| **HA** | **NA** | **Isolate name** |
| --- | --- | --- |
| H10 | N1 | A/Mallard/Sweden/102087/2009/H10N1 |
| H6 | N2 | A/Mallard/Sweden/99825/2009 |
| H9 | N2 | A/Mallard/Sweden/99668/2009 |
| H9 | N2 | A/Chicken/Egypt/S12568C/2016/NA/H9N2 |
| H16 | N3 | A/Black-headed Gull/Sweden/74340/2008 |
| H8 | N4 | A/Mallard/Sweden/58256/2006 |
| H15 | N5 | A/Mallard/Sweden/139647/2012 |
| H4 | N6 | A/Mallard/Sweden/80148/2008 |
| H7 | N7 | A/Mallard/Sweden/124987/2010 |
| H3 | N8 | A/Mallard/Sweden/101487/2009 |
| H11 | N9 | A/Mallard/Sweden/102103/2009 |

Supplementary Table 2. List of primers used for cloning the wild-bird derived NA-segments (N1-N9).

| **Primers for cloning NA segments** | | |
| --- | --- | --- |
| **Virus** | **Primer** | **Restriction site** |
| H10N1 | **Fw:** GATCGGTCTCAGGGAGCAAAAGCAGGAGTTCAAAATGA  **Rv**: GATCGGTCTCGTATTAGTAGAAACAAGGAGTTTTTTGAA | BsaI |
| H6N2 | **Fw:** GATCCGTCTCAGGGAGCGAAAGCAGGAGTGAAAATG  **Rv:** GATCCGTCTCGTATTAGTAGAAACAAGGAGTTTTTTTCTA | BsmBI |
| mH9N2 | **Fw:** GATCCGTCTCAGGGAGCGAAAGCAGGAGTGAAAATG  **Rv:** GATCCGTCTCGTATTAGTAGAAACAAGGAGTTTTTTTC | BsmBI |
| H16N3 | **Fw:** GATCCGTCTCAGGGAGCAAAAGCAGGTGTGAAATGAATC  **Rv:** GATCCGTCTCGTATTAGTAGAAACAAGGTGTTTTTTCTATTAC | BsmBI |
| H8N4 | **Fw:** GATCCGTCTCAGGGAGCGAAAGCAGGAGTTTCATAATG  **Rv:** GATCCGTCTCGTATTAGTAGAAACAAGGAGTTTTTTCG | BsmBI |
| H15N5 | **Fw:** GATCCGTCTCAGGGAGCAAAAGCAGGAGTTTAAAATGAATCC  **Rv:** GATCCGTCTCGTATTAGTAGAAACAAGGAGTTTTTTCACAAGC | BsmBI |
| H4N6 | **Fw:** GATCCGTCTCAGGGAGCAAAAGCAGGGTGAAAATGAATCC Rv: GATCCGTCTCGTATTAGTAGAAACAAGGGTGTTTTTCCT | BsmBI |
| H7N7 | **Fw:** GATCCGTCTCAGGGAGCAAAAGCAGGGTGATTGAGAATG  **Rv:** GATCCGTCTCGTATTAGTAGAAACAAGGGTGTTTTTGCA | BsmBI |
| H3N8 | **Fw:** GATCCGTCTCAGGGAGCAAAAGCAGGAGTTTAAAATGAATC  **Rv:** GATCCGTCTCGTATTAGTAGAAACAAGGAGTTTTT | BsmBI |
| H11N9 | **Fw:** GATCCGTCTCAGGGAGCAAAAGCAGGGTCAAGATGAATC  **Rv:** GATCCGTCTCGTATTAGTAGAAACAAGGGTCTTTTTTGC | BsmBI |
| **Primers for site-directed mutagenesis** | | |
| **Virus** | **Primer** |  |
| H10N1 | **Fw:** AGCAATACCAATTTTCTTG  **Rv:** GCATGATTCAGGCTGGTATT |  |
| H6N2 | **Fw:** AACACCACCATAGAAAAAGAAC  **Rv:** TTGATTGTTTGAGGGGATGC |  |
| mH9N2 | **Fw:** AATACTATCATAGAGAAAGAAC  **Rv:** TTGATTGTTCGAGGGGATGC |  |
| chH9N2 | **Fw:** GGTACTATCATAGAGAAGGAAATTTG  **Rv:** TTGATTTTTCGAGATGTTTGTGTACTCG |  |
| H16N3 | **Fw:** ATCAACAACATAACAAATG  **Rv:** GTTTGGTGCAATTTCACC |  |
| H8N4 | **Fw:** TATACCACTATAACTGAGCCCTCAAGC  **Rv:** CGAACAGGGTTGTTCGCTTGTTTGAG |  |
| H15N5 | **Fw:** ATAGGAAGGGAATCGACCCACGAACCCG  **Rv:** CTCAGTTGTGTTGCATGTGTATGCCTTG |  |
| H4N6 | **Fw:** AATATTACTAACATTATAG  **Rv:** GCTCGGAATGTTCATGTCTG |  |
| H7N7 | **Fw:** ACAATAATTACCAAGGAAAC  **Rv:** CGTTAAATTCTCCTCCTG |  |
| H3N8 | **Fw:** GTAGTCGAGTACGTGCCGTATTGGAATG  **Rv:** TATTGTTTCGTTGCAGACCCCATTGTTTC |  |
| H11N9 | **Fw:** ATAAGTAACACCAACATCCAAATGGAAG  **Rv:** TTCAGGTTGTGAGTGTGAGCAATTGCAG |  |
| **Primers for qPCR confirmation** | | |
| **Neuraminidase** | **Primer** |  |
| N1 | **Fw:** GRCCTTGYTTCTGGGTKGA  **Rv:** ACCGTCTGGCCAAGACCA  **Probe:** CAATYTGGACYAGTGGRAGYAGCAT |  |
| N2 | **Fw:** CAGAGTRTGGTGGACITC  R**v:** TTGCGAAAGCTTAYATNGVCAT  **Probe:** CATCAGGCCATGAGCCTGTYCCAT |  |
| N3 | **Fw:** GCAAYAGTATAGTTACYTTCTG  **Rv:** TTACTTGGGCATRAACCCAAT  **Probe:** AGACAATGAACCTGGATCGGGVAA |  |
| N4 | **Fw:** GACTAGYGGTAGTAGYATTGC  **Rv:** CGAAAAATYACTTGTCTATGTCAA  **Probe:** TGGTCRTGGCCYGATGGCGCTCT |  |
| N5 | **Fw:** CCTTCAGAATGCAGRACYTT  **Rv:** TAGCAGACCAYCCRACGGA  **Probe:** TAATGAGCGTRCCATTGGGATCCTC |  |
| N6 | **Fw:** GGTGAMAATGAAYCCAAAYCA  **Rv:** CTTRTARTGRAGTCCGATGTT  **Probe:** CATYTCAGCIACAGGARTRACACTATC |  |
| N7 | **Fw:** GTTGAATTAATWAGAGGAAGRCC  **Rv:** GATYTGTGCCCCATCRGGGA  **Probe:** CCTATGTGGRAGCCCATTCCCAGT |  |
| N8 | **Fw:** CTGATCTCTCTTACAGGGTTG  **Rv:** GCTCCATCRTGCCAYGACCA  **Probe:** TCHAGYAGCTCCATTGTRATGTGTGGAGT |  |
| N9 | **Fw:** AGYATAGTATCRATGTGTTCCAG  **Rv:** GTACTCTATTYTAGCCCCRTC  **Probe:** TTCCTRGGACAATGGRACTGGCC |  |

Supplementary Table 3. List of p-values generated from Tukey multiple comparison of the replication kinetics data, comparing the mean TCID_50_ of rH9Nx with intact neuraminidase in DF-1 and A549 cells. P-values less than 0.05 were considered significant. Bold p-values indicate significantly higher mean TCID_50_ in A549 cells compared to DF-1. Underlined p-values indicate significantly lower TCID_50_ in A549 cells compared to DF-1. Non-significant comparisons are denoted ‘ns’.

|  | 8h | 24h | 48h | 72h |
| --- | --- | --- | --- | --- |
| **chH9N2** | ns | 0,0007 | 0,0136 | 0,0447 |
| **rH9N1** | ns | ns | ns | <0,0001 |
| **rH9N2_(H6)_** | ns | 0,0121 | ns | ns |
| **rH9N2_(H9)_** | ns | 0,0156 | <0,0001 | 0,0013 |
| **rH9N3** | 0,0024 | ns | 0,0035 | 0,0032 |
| **rH9N4** | **0,0284** | 0,0057 | 0,0144 | ns |
| **rH9N5** | ns | ns | ns | 0,0381 |
| **rH9N6** | <0,0001 | 0,0005 | 0,0011 | 0,0080 |
| **rH9N7** | ns | ns | ns | 0,0292 |
| **rH9N8** | ns | ns | **0,0267** | ns |
| **rH9N9** | ns | ns | ns | ns |

Supplementary Table 4. List of p-values generated from Tukey multiple comparison of the replication kinetics data, comparing the mean TCID_50_ of rH9Nx with neuraminidase stalk-deletion in DF-1 and A549 cells. P-values less than 0.05 were considered significant. Bold p-values indicate significantly higher mean TCID_50_ in A549 cells compared to DF-1. Underlined p-values indicate significantly lower TCID_50_ in A549 cells compared to DF-1. Non-significant comparisons are denoted ‘ns’

|  | 8h | 24h | 48h | 72h |
| --- | --- | --- | --- | --- |
| **chH9N2_Δ_** | ns | 0,0016 | 0,0067 | 0,0357 |
| **rH9N1_Δ_** | ns | ns | ns | 0,0135 |
| **rH9N2_(H6) Δ_** | ns | 0,0012 | 0,0024 | <0,0001 |
| **rH9N2_(H9) Δ_** | ns | ns | ns | 0,0213 |
| **rH9N3_Δ_** | ns | 0,0105 | 0,0014 | 0,0034 |
| **rH9N4_Δ_** | 0,0027 | 0,0018 | 0,0140 | 0,0044 |
| **rH9N5_Δ_** | ns | ns | ns | 0,0005 |
| **rH9N6_Δ_** | ns | <0,0001 | 0,0101 | 0,0005 |
| **rH9N7_Δ_** | ns | ns | ns | 0,0458 |
| **rH9N8_Δ_** | ns | **0,0002** | ns | ns |
| **rH9N9_Δ_** | ns | ns | ns | 0,0020 |

Supplementary Table 5. List of p-values generated from Tukey multiple comparison of the replication kinetics data, comparing the mean TCID_50_ of rH9Nx with stalk-deletion replication in both DF-1 cells and A549 with rH9Nx with intact neuraminidase. P-values less than 0.05 were considered significant. Bold p-values indicate significantly higher mean TCID_50_ value at a given timepoint. Underlined p-values indicate significantly lower TCID_50_ at a given timepoint. Non-significant comparisons are denoted ‘ns’.

|  | DF-1 | | | |  | A549 | | | |
| --- | --- | --- | --- | --- | --- | --- | --- | --- | --- |
|  | 8h | 24h | 48h | 72h |  | 8h | 24h | 48h | 72h |
| **chH9N2_Δ_** | ns | ns | ns | ns | **chH9N2_Δ_** | ns | ns | ns | ns |
| **rH9N1_Δ_** | ns | ns | 0,0067 | ns | **rH9N1_Δ_** | ns | ns | ns | ns |
| **rH9N2_(H6) Δ_** | 0,0203 | 0,0390 | 0,0055 | ns | **rH9N2_(H6) Δ_** | 0,0484 | 0,0001 | 0,0028 | 0,0169 |
| **rH9N2_(H9) Δ_** | 0,0061 | 0,0454 | 0,0003 | 0,0011 | **rH9N2_(H9) Δ_** | ns | ns | ns | ns |
| **rH9N3_Δ_** | ns | ns | ns | ns | **rH9N3_Δ_** | 0,0036 | ns | 0,0047 | 0,0175 |
| **rH9N4_Δ_** | ns | ns | 0,0254 | ns | **rH9N4_Δ_** | 0,0017 | 0,0306 | 0,0108 | 0,0058 |
| **rH9N5_Δ_** | ns | ns | ns | ns | **rH9N5_Δ_** | ns | 0,0002 | ns | 0,0339 |
| **rH9N6_Δ_** | ns | 0,0477 | 0,0240 | 0,0075 | **rH9N6_Δ_** | **0,0003** | **0,0372** | **0,0072** | ns |
| **rH9N7_Δ_** | ns | ns | 0,0008 | 0,0036 | **rH9N7_Δ_** | ns | 0,0297 | 0,0286 | 0,0001 |
| **rH9N8_Δ_** | ns | ns | ns | ns | **rH9N8_Δ_** | ns | **0,0003** | ns | ns |
| **rH9N9_Δ_** | **0,0103** | ns | **0,0079** | **0,0334** | **rH9N9_Δ_** | **0,0018** | **0,0413** | **0,0172** | **0,0334** |

Supplementary Table 6. List of p-values generated from Tukey multiple comparison of the replication kinetics data, comparing the mean TCID_50_ between rH9Nx with neuraminidase stalk-deletion and parental chH9N2 virus in MDCK-ll cells. P-values less than 0.05 were considered significant. Bold p-values indicate significantly higher mean TCID_50_ value at a given timepoint. Underlined p-values indicate significantly lower TCID_50_ at a given timepoint. Non-significant comparisons are denoted ‘ns’.

|  | MDCK-ll | | | |
| --- | --- | --- | --- | --- |
|  | 8h | 24h | 48h | 72h |
| **rH9N1** | 0,0160 | 0,0336 | ns | **0,0216** |
| **rH9N2_(H6)_** | 0,0241 | 0,0069 | ns | ns |
| **rH9N2_(H9)_** | ns | ns | ns | **0,0325** |
| **rH9N3** | 0,0223 | 0,0174 | ns | **0,0453** |
| **rH9N4** | ns | 0,0411 | ns | ns |
| **rH9N5** | ns | ns | ns | ns |
| **rH9N6** | 0,0024 | 0,0027 | 0,0106 | 0,0015 |
| **rH9N7** | 0,0019 | 0,0053 | ns | ns |
| **rH9N8** | ns | **0,0207** | ns | ns |
| **rH9N9** | ns | 0,0058 | ns | ns |
| **chH9N2_Δ_** | ns | 0,0399 | ns | ns |
| **rH9N1_Δ_** | ns | 0,0312 | ns | ns |
| **rH9N2_(H6) Δ_** | ns | ns | ns | ns |
| **rH9N2_(H9) Δ_** | ns | 0,0194 | ns | ns |
| **rH9N3_Δ_** | 0,0091 | ns | 0,0138 | ns |
| **rH9N4_Δ_** | ns | ns | ns | 0,0185 |
| **rH9N5_Δ_** | **0,0269** | ns | ns | 0,0364 |
| **rH9N6_Δ_** | ns | ns | ns | ns |
| **rH9N7_Δ_** | **0,0033** | ns | ns | 0,0015 |
| **rH9N8_Δ_** | **0,0114** | ns | ns | ns |
| **rH9N9_Δ_** | ns | ns | ns | ns |

Supplementary Table 7. List of p-values generated from Tukey multiple comparison of the replication kinetics data, comparing rH9Nx with and without neuraminidase stalk deletion in MDCK-ll . P-values less than 0.05 were considered significant. Bold p-values indicate significantly higher mean TCID_50_ value at a given timepoint. Underlined p-values indicate significantly lower TCID_50_ at a given timepoint. Non-significant comparisons are denoted ‘ns’.

|  | MDCK-ll | | | |
| --- | --- | --- | --- | --- |
|  | 8h | 24h | 48h | 72h |
| **chH9N2_Δ_** | ns | ns | ns | ns |
| **rH9N1_Δ_** | ns | ns | ns | 0,0235 |
| **rH9N2_(H6) Δ_** | ns | ns | ns | ns |
| **rH9N2_(H9) Δ_** | ns | ns | 0,0095 | 0,0004 |
| **rH9N3_Δ_** | 0,0161 | ns | 0,0049 | ns |
| **rH9N4_Δ_** | **0,0035** | ns | 0,0058 | 0,0012 |
| **rH9N5_Δ_** | **0,0270** | ns | ns | 0,0045 |
| **rH9N6_Δ_** | **<0,0001** | **<0,0001** | **0,0481** | **0,0056** |
| **rH9N7_Δ_** | **<0,0001** | ns | ns | 0,0021 |
| **rH9N8_Δ_** | ns | 0,0233 | 0,0437 | 0,0116 |
| **rH9N9_Δ_** | **<0,0001** | **0,0083** | ns | 0,0329 |

Supplementary Table 8. List of p-values generated from Tukey multiple comparison of the NA-activity data, comparing the relative fluorescence (RLU) of rH9Nx viruses and chH9N2. P-values less than 0.05 were considered significant. Bold p-values indicate significantly higher mean RLU.. Underlined p-values indicate significantly lower mean RLU. Non-significant comparisons are denoted ‘ns’.

|  | **16 HAU** | **8 HAU** | **4 HAU** | **2 HAU** | **1 HAU** | **0.5 HAU** | **0.25 HAU** | **0.125 HAU** |
| --- | --- | --- | --- | --- | --- | --- | --- | --- |
| **rH9N1** | 0,0082 | 0,0288 | 0,0102 | 0,0122 | ns | ns | ns | ns |
| **rH9N2_(H6)_** | <0,0001 | <0,0001 | <0,0001 | 0,0028 | ns | ns | ns | ns |
| **rH9N2_(H9)_** | <0,0001 | <0,0001 | <0,0001 | 0,0001 | 0,0446 | ns | ns | ns |
| **rH9N5** | <0,0001 | <0,0001 | 0,0008 | 0,0315 | ns | ns | ns | ns |
| **rH9N6** | <0,0001 | <0,0001 | <0,0001 | <0,0001 | <0,0001 | 0,0029 | 0,0024 | ns |
| **rH9N7** | <0,0001 | 0,0009 | 0,0146 | ns | ns | ns | ns | ns |
| **rH9N8** | <0,0001 | <0,0001 | <0,0001 | <0,0001 | <0,0001 | <0,0001 | 0,0466 | ns |
| **rH9N9** | <0,0001 | <0,0001 | <0,0001 | <0,0001 | 0,0403 | ns | ns | ns |
| **chH9N2_Δ_** | <0,0001 | <0,0001 | <0,0001 | <0,0001 | 0,0262 | ns | ns | ns |
| **rH9N1_Δ_** | ns | ns | ns | ns | ns | ns | ns | ns |
| **rH9N2_(H6)Δ_** | <0,0001 | <0,0001 | 0,0015 | 0,0433 | ns | ns | ns | ns |
| **rH9N2_(H9) Δ_** | <0,0001 | <0,0001 | <0,0001 | 0,0039 | ns | ns | ns | ns |
| **rH9N3_Δ_** | ns | ns | ns | ns | ns | ns | ns | ns |
| **rH9N4_Δ_** | ns | ns | ns | ns | ns | ns | ns | ns |
| **rH9N5_Δ_** | <0,0001 | <0,0001 | <0,0001 | 0,0007 | ns | ns | ns | ns |
| **rH9N6_Δ_** | <0,0001 | <0,0001 | <0,0001 | <0,0001 | <0,0001 | 0,0075 | ns | ns |
| **rH9N7_Δ_** | ns | ns | ns | ns | ns | ns | ns | ns |
| **rH9N8_Δ_** | <0,0001 | <0,0001 | <0,0001 | <0,0001 | <0,0001 | <0,0001 | 0,0375 | ns |
| **rH9N9_Δ_** | <0,0001 | <0,0001 | <0,0001 | 0,0003 | ns | ns | ns | ns |

Supplementary Table 9. List of p-values generated from Tukey multiple comparison of the NA-activity data, comparing the relative fluorescence (RLU) of rH9Nx with and without neuraminidase stalk deletions. P-values less than 0.05 were considered significant. Bold p-values indicate significantly higher mean RLU. Underlined p-values indicate significantly lower mean RLU. Non-significant comparisons are denoted ‘ns’

|  | **16 HAU** | **8 HAU** | **4 HAU** | **2 HAU** | **1 HAU** | **0.5 HAU** | **0.25 HAU** | **0.125 HAU** |
| --- | --- | --- | --- | --- | --- | --- | --- | --- |
| **chH9N2_Δ_** | ns | ns | ns | ns | ns | ns | ns | ns |
| **rH9N1_Δ_** | **<0,0001** | **0,0002** | **0,0096** | **0,0484** | ns | ns | ns | ns |
| **rH9N2_(H6) Δ_** | **0,0003** | **0,0089** | ns | ns | ns | ns | ns | ns |
| **rH9N2_(H9) Δ_** | 0,0132 | ns | ns | ns | ns | ns | ns | ns |
| **rH9N3_Δ_** | **0,0056** | **0,0060** | **0,0121** | ns | ns | ns | ns | ns |
| **rH9N4_Δ_** | ns | ns | ns | ns | ns | ns | ns | ns |
| **rH9N5_Δ_** | 0,0042 | 0,0031 | ns | ns | ns | ns | ns | ns |
| **rH9N6_Δ_** | **<0,0001** | **<0,0001** | ns | ns | ns | ns | ns | ns |
| **rH9N7_Δ_** | **0,0031** | **0,0327** | ns | ns | ns | ns | ns | ns |
| **rH9N8_Δ_** | ns | ns | 0,0462 | ns | ns | ns | ns | ns |
| **rH9N9_Δ_** | ns | ns | ns | ns | ns | ns | ns | ns |
